# Effect of Monotherapy and Combination Therapy of Pantoprazole and Aprepitant in Gastric Esophageal Reflux Disease in Albino Rats

**DOI:** 10.1155/2014/183147

**Published:** 2014-03-23

**Authors:** Kamleshwar Shukla, Prince Raj, Arun Kumar, Mukesh Kumar, Gaurav Kaithwas

**Affiliations:** ^1^Department of Pharmaceutical Sciences, Babasaheb Bhimrao Ambedkar University, Vidya Vihar, Raebareli Road, Lucknow 226 025, India; ^2^Department of Applied Animal Sciences, Babasaheb Bhimrao Ambedkar University, Vidya Vihar, Raebareli Road, Lucknow 226 025, India

## Abstract

The present study was undertaken to elucidate the effect of pantoprazole and aprepitant on experimental esophagitis in albino rats. Groups of rats, fasted overnight, received normal saline (3 mL/kg, sham control) or toxic control (3 mL/kg) or pantoprazole (30 mg/kg) or aprepitant (10 mg/kg), or their combinations and were subjected to pylorus and forestomach ligation. Animals were sacrificed after 8 h and evaluated for the gastric pH, volume of gastric juices, total acidity, esophagitis index, and free acidity. Esophageal tissues were further subjected to estimations of TBARS, GSH, catalase, and SOD. Treatment with pantoprazole and aprepitant significantly inhibited the gastric secretion, total acidity, and esophagitis index. The treatment also helped to restore the altered levels oxidative stress parameters to normal.

## 1. Introduction

Gastroesophageal reflux disease (GERD) is a gastrointestinal disorder and defined as a condition that develops due to reflux of stomach content into esophagus, causing troublesome symptoms or complications [1]. Heartburn is the most common symptom of GERD and is estimated to occur daily in seven percent of the US population [2]. In addition to heartburn, regurgitation and difficulty in swallowing are common GERD symptoms. GERD also includes subcategories of diagnosis: nonerosive esophageal reflux disease (NERD) and the other pathologies that result due to progression of GERD, including esophageal ulcer, esophageal stricture, Barrett's esophagus, and Barrett's carcinoma [1]. Several lines of treatments exist for the treatment and clinical management of GERD including proton pump inhibitors (PPIs) and H_2_ blockers. The clinical management of GERD is difficult and requires long-term therapy due to relapsing nature of disease. The clinical management by PPIs and H_2_ blockers is not very effective due to weak inhibitory activity in early phase and less effectiveness of the therapy within the initial hours of dosing [3]. From above it became obvious that GERD is a chronic disease and requires long-term symptomatic and pathological management [4].

Aprepitant is a selective high affinity antagonist of human substance P/neurokinin (NK1) receptor. Aprepitant has little or no affinity for serotonin (5-HT3), dopamine, and corticosteroid receptor and is used against chemotherapy-induced nausea and vomiting (CINV) and postoperative nausea and vomiting (PONV). In the antecedent studies aprepitant has demonstrated the inhibition of emesis induced by cytotoxic chemotherapeutic agents, such as cisplatin, via central action.

PPIs are used for the treatment of condition such as ulcer and Zollinger-Ellison syndrome that are caused by stomach acid [5]. Pantoprazole like other proton-pump inhibitors is the most potent gastric acid suppressants because of their ability to inhibit the proton pump H^+^-K^+^-ATPase, which is the final common pathway of gastric acid secretion and blocks the enzyme in the wall of the stomach that produces acid. It suppresses nocturnal and day time as well as food-stimulated gastric acid secretion by blocking the enzyme; the production of acid is decreased, and this allows the stomach and esophagus to heal [6].

In view of above, we hypothesize that aprepitant by virtue of its NK1 receptor blocking action (e.g., antiemetic action) and pantoprazole through proton pump inhibitor action will provide a long-term systemic relief in management of GERD. Henceforth, the present study has been undertaken with the objective to cut down the reflux and to decrease the production of gastric content using combination therapy of aprepitant and pantoprazole.

## 2. Methods and Materials

### 2.1. Drug and Chemicals

Aprepitant was received as a gift sample from Glanemark Pharmaceuticals Ltd. Mumbai, India, and pantoprazole was procured from the local market. All other chemicals were used of analytical grade.

### 2.2. Animals

Albino Wistar rats (120–150 gm) were obtained from the animal house of BBDNIIT, Lucknow. The albino rats were kept in polypropylene cage under standard condition of temperature (37 ± 1°C) with 12 h light: dark cycle with free access to a commercial pellet diet and water [7]. The experimental protocol was approved by Institutional Animal Ethics Committee (IAEC) of BBDNIIT, Lucknow (Ref. number BBDNIIT/IAEC/11/2012).

### 2.3. Induction of Esophagitis

**Figure 1:**
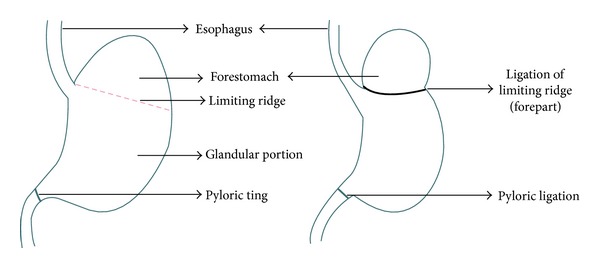
Schematic representation of pylorus and forestomach ligation.

Animals were randomized and divided into five groups of six animals each. Groups of rats, fasted overnight, received normal saline or control vehicle (0.9% NaCl in double distilled water), pantoprazole, aprepitant, or their combination as described in Table 2. After 1 h, coeliotomy was performed and esophagitis was induced by ligating the forestomach and pylorus with 2-0 silk suture, under pentobarbitone anesthesia [8] (Figure 1).

After 8 h animals were sacrificed by cervical dislocation and the chest was opened with a median incision and the tissue esophagus and the stomach were removed. The stomach was opened along the greater curvature and the esophagus was dissected out by extending the dissection line along the major axis. The tissue was washed with normal saline and examined for lesion. The severity of the erosions was scored using Table 1 and the index was calculated by dividing the total score by ten, which was designated as esophagitis index [9]. The volume of gastric juice, total acidity, and pH was measured as described subsequently under “Gastric secretion in pylorus ligated rats” [10].

### 2.4. Scanning Electron Microscopy for Esophageal Tissue

Samples were fixed in 2.5% glutaraldehyde for 6 h at 4°C and washed in 0.1 M phosphate buffer, for 3 changes each of 15 min at 4°C. 1% osmium tetroxide was used as a postfixation for 2 h at 4°C and samples were washed in 0.1 M phosphate buffer for 3 changes each of 15 min at 4°C to remove the uncreative fixative. Specimens were dehydrated by using increasing concentration of acetone, namely, 30%, 50%, 70%, 90%, 95%, and 100% (dry acetone), to remove water at 4°C for 30 min period. After that, specimens were air-dried (critical point, i.e., 31.5 at 1100 psi). Specimens were mounted on to the aluminium stub with conductive paint or adhesive tape. Then specimens were observed in scanning electron microscope (JEOL-JSM-6490LV).

### 2.5. Statistical Analysis

All data were presented as mean ± SD and analyzed by one-way ANOVA followed by Bonferroni test for the possible significance identification between the various groups. *P* < 0.05, *P* < 0.01, and *P* < 0.001 were considered statistically significant. Statistical analysis was carried out using GraphPad software (3.2), San Diego, CA.

## 3. Results

Oral administration of aprepitant (10 mg/kg) significantly inhibited the esophagitis in the albino rats. Aprepitant significantly inhibited the esophagitis index (53.54%), gastric volume (39.37%), free acidity (33.69%), and total acidity (29.32%) in comparison with toxic control. Pantoprazole produced 80.64% inhibition of esophagitis index, respectively. The oral administration of aprepitant in combination of pantoprazole markedly decreased the gross volume of gastric juices (45.63%), total acidity (40.45%), free acidity (16.22%), and esophagitis index (62.58%) in comparison to toxic control (Table 2).

**Figure 2:**
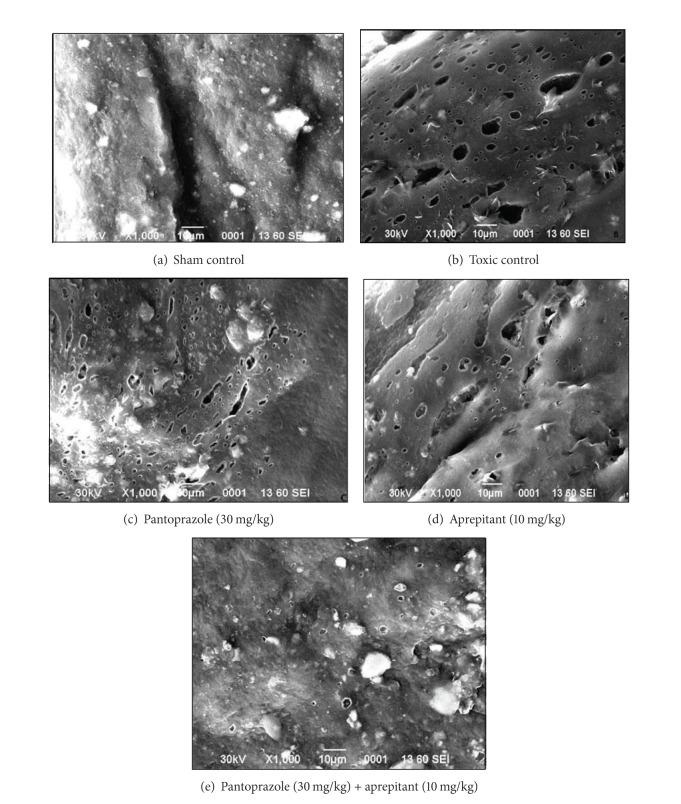
Scanning electron microscopic photomicrographs of the esophageal tissues.

Tissue MDA level in the control and toxic control was found to be 0.81 ± 0.08 and 7.50 ± 0.52, respectively. There was significant decrease in the MDA level in the combination treatment in comparison to monotherapy and control as well. Blood GSH level in the normal control was found to be 208.60 ± 3.83 and in toxic control was 74.20 ± 7.85; similarly tissue catalase level in the control was found to be 24.49 ± 2.19 and in toxic control was 7.02 ± 1.72. There was significant increase in the blood GSH level and catalase level in the combination therapy in comparison to monotherapy. Tissue SOD level in the control was found to be 5.32 ± 0.27 and in toxic control was 0.89 ± 0.073. There was significant increase in the SOD level in the combination therapy in comparison to monotherapy (Table 3). The above results were also supported by the SEM photomicrograph (Figure 2).

## 4. Discussion

Ligating the forestomach and pylorus developed reflux esophagitis in all the animals marked by macroscopically visible necrosis and significant ulceration in the esophagus. Treatment with pantoprazole and aprepitant significantly inhibited the ulcer formation in esophagus. Treatment with aprepitant demonstrated significant protection against the reflux esophagitis in the experimental animals. Aprepitant significantly reduced the esophagitis index, gastric volume, free acidity, and total acidity in comparison with toxic control. The beneficial effects of aprepitant observed in the present study could be attributed to its NK_1_ receptor antagonistic action providing antiemetic property to the aprepitant. The same has been attributed for its use in the management of chemotherapy induced nausea and vomiting [11]. The NK_1_ receptor blocking action of the aprepitant inhibits the reflux of acid into the esophagus and thereby provides a symptomatic relief in the present experiment.

Pantoprazole is a proton pump inhibitor having an H^+^K^+^ ATPase and carbonic anhydrase inhibitory activity [12]. Pantoprazole is a well-established drug used for the treatment of peptic ulcer, which inhibits the secretion from the gastric cells and helps in providing relief in reflux disease and mucosal curing in gastric ulcers and GERD [13]. In the present experiment pantoprazole demonstrated reduction in gastric volume, total acidity, and esophagitis index and this observation is in concordance with the previous studies.

The combination of pantoprazole and aprepitant inhibited the esophagitis index, decreased the volume of gastric juices, and reduced the pH to a significant level, suggesting the possible synergistic effect. Thus the effect against GERD could be congregately attributed to the antisecretory action of pantoprazole and antiemetic action by aprepitant, and the same seems to accounts for decrease in gross volume of gastric juice secretion, total acidity, and esophagitis index in the present experiment as well.

Previous studies have elaborated the role of free radicals in pathogenesis of the reflux esophagitis in experimental animals [14]. Reflux esophagitis has been reported to increase malondialdehyde, a stable product of lipid peroxidation and a sensitive marker of membrane damage in esophageal tissues [15, 16]. The oxidative stress leads to degradation of cellular membrane which produces MDA, which is a reactive substance and forms a color complex with the thiobarbituric acid. Significant increase in MDA in the toxic control suggests active participation of reactive oxygen species (ROS) and oxidative stress in GERD. This further enumerates that the ligation of pylorus and forestomach generates free radical species that attack lipid components, leading to lipid peroxidation. It is pertinent to mention that the concomitant administration of the pantoprazole and aprepitant as a monotherapy and combination therapy significantly inhibited the lipid peroxidation evidenced by decreased formation of MDA.

The glutathione (GSH) is a ubiquitous tripeptide, which is the most abundant low molecular weight thiol in almost all cells and is involved in a wide range of enzymatic reaction. A major function of GSH is to serve as a reductant in oxidation reduction processes, a function resulting in the formation of glutathione disulfide (GSSG). Free radical damage leads to consumption of GSH in the first few hours of oxidative stress, directing decreased GSH level, a marker of short-term oxidative stress, and treatment with pantoprazole and aprepitant has significantly helped restore the same [17, 18]. Decrease in the levels of GSH represents its increased utilization by the cells due to oxidative stress and treatment with pantoprazole and aprepitant alone or in combination has significantly helped restore the levels of GSH; this effect could be attributed either due to increased oxidative stress or increased biogenesis of GSH. It would be worthwhile to mention that combination therapy of pantoprazole and aprepitant exhibited maximum antioxidant effect in comparison to monotherapy.

SOD is a free radical scavenging enzyme which neutralizes superoxide free radicals generated during the metabolism of drug; hence its concentration in the tissue decreases with increase in the time. SOD serves a key antioxidant role and decreases oxidative stress in the experimental animals; SOD scavenges the H_2_O_2_ to form water and molecular oxygen; the process involves the formation of hydroxyl and molecular oxygen free radical as the intermediate products [19]. The SOD in conjugation with catalase constitutes the major defense against free radicals. Catalase is a hemeprotein which catalyses the reduction of hydrogen peroxide (produced due to scavenging effect of SOD) and protect the tissue from highly reactive hydroxyl radical [20, 21]. In the present experiment we observed simultaneous decrease in SOD and catalase activity after the ligation of pyloric end and forestomach. This decrease in SOD and catalase could be attributed to the increased oxidative stress and thereby increased utilization. Treatment with pantoprazole and aprepitant restored the diminished levels of SOD and catalase suggesting the decreased oxidative stress. It would be pertinent to mention that the combination therapy observed better antioxidant activity in comparison to monotherapy. The above observed findings are also supported by the finding from scanning electron microscopy.

It would be worthwhile to mention that no untoward effect was observed in animals treated with pantoprazole and aprepitant either alone or in combination. Our results suggest the possible therapeutic potential of combination therapy of pantoprazole and aprepitant against reflux esophagitis in experimental animals without any untoward effect. However, further studies are required to be undertaken to confirm its therapeutic potential against GERD.

## Figures and Tables

**Table 1:** Scoring of erosion and severity.

Erosion (mm)	1 or less	1-2	2-3	>3
Score	1	2	3	4

**Table 2:** Effect of pantoprazole and aprepitant as monotherapy and combination therapy on gastric pH, volume of gastric juice, total acidity, free acidity, and esophagitis index.

S. number	Treatment (p.o)	pH	Volume of gastric juices (mL/100 g)	Total acidity (mEq/L)	Esophagitis index	Free acidity (meq/L)
Group-I	Sham control (normal saline, 3 mL/kg,)	3.27** ± **0.09	2.39 ± 0.07	30.60** ± **0.48	0.16 ± 0.02	25.86** ± **1.22

Group-II	Toxic control (normal saline, 3 mL/kg, )	2.00** ± **0.09***	3.20 ± 0.04***	55.38 ± 4.56***	3.10 ± 0.05***	42.80 ± 1.45***

Group-III	Pantoprazle (30 mg/kg)	4.00 ± 0.06***	1.68 ± 0.07*** (47.50%)	30.74** ± **3.74 (44.49%)	0.60 ± 0.02*** (80.65%)	27.14 ± 0.55 (36.59%)

Group-IV	Aprepitant (10 mg/kg)	3.28 ± 0.08	1.94 ± 0.05*** (39.38%)	39.14** ± **2.05*** (29.32%)	1.44 ± 1.08*** (53.55%)	28.38 ± 0.78** (33.69%)

Group-V	Pantoprazole + aprepitant (30 mg/kg + 10 mg/kg)	3.42 ± 0.07*	1.74 ± 0.05*** (45.63%)	32.98 ± 3.09 (40.45%)	1.16 ± 0.32*** (62.58%)	26.58 ± 0.51 (16.22%)

Each group contains six animals. Values are represented as mean ± SD. Statistical significance compared to toxic control using one-way ANOVA followed by Bonferroni test (*P < 0.05, **P < 0.01, and ***P < 0.001). Values in parenthesis represent percentage inhibition.

**Table 3:** Effect of pantoprazole and aprepitant as monotherapy and combination therapy on blood GSH, TBARS, SOD, and catalase in esophageal tissue.

S. number	Treatment (p.o.)	SOD (SOD/mg of protein)	TBARS (nM of MDA/mg of protein)	Catalase (nM of H_2_O_2_/min/mg of protein)	Blood GS H (mg%)
Group-I	Sham control (normal saline, 3 mL/kg)	5.32 ± 0.26	0.81 ± 0.08	24.49 ± 2.18	208.60 ± 3.83

Group-II	Toxic control (normal saline, 3 mL/kg)	0.89 ± 0.07***	7.50 ± 0.52***	7.02 ± 1.72***	74.20 ± 7.85***

Group-III	Pantoprazole (30 mg/kg)	3.16 ± 0.38***	3.06 ± 0.54***	18.01 ± 1.06***	141.00 ± 7.56***

Group-IV	Aprepitant (10 mg/kg)	3.72 ± 0.07***	2.10 ± 0.22***	22.00 ± 0.57	181.20 ± 10.04**

Group-V	Pantoprazole + aprepitant (30 mg/kg + 10 mg/kg)	4.50 ± 0.04***	1.06 ± 0.34	24.40 ± 0.97	194.20 ± 20.76

Each group contains six animals. Values are represented as mean ± SD. Statistical significance compared to toxic control using one-way ANOVA followed by Bonferroni test (*P < 0.05, **P < 0.01, and ***P < 0.001).

## Abbreviations

GERD: Gastroesophageal reflux disease
GSH: Glutathione
PPIs: Proton pump inhibitors
TBARS: Thiobarbituric acid reactive substance
MDA: Malondialdehyde
SOD: Superoxide dismutase.
